# Editorial: Aerospace health and safety: today and the future, volume I

**DOI:** 10.3389/fpubh.2023.1264524

**Published:** 2023-09-25

**Authors:** Christopher Scheibler, Mardi A. Crane-Godreau, Eileen McNeely

**Affiliations:** ^1^School of Public Health, Harvard University, Boston, MA, United States; ^2^Independent Researcher, Arlington, VT, United States; ^3^Department of Environmental Health, School of Public Health, Harvard University, Boston, MA, United States

**Keywords:** aerospace medicine, flight environment, inflight medical emergencies, aviation health, human factors, circadian rhythm disruption, cosmic ionizing radiation, space travel

Over the last few years, the world has experienced significant public health challenges due to the arrival of the SARS-CoV-2 virus and related pandemic effects. Similar to most other major industries, there have been substantial impacts to aviation. When this topic initially launched in 2019, an estimated 4.1 billion passengers flew annually (1), however the current decreased passenger volume is not expected to return to 2019 levels until 2024 (2).

Commercial air travel experienced an initial decrease in volume with subsequent employee layoffs followed by returning travel demand complicated by a reduced labor force. The industry has experienced multiple pendulum swings initiated by pandemic waves with variable impacts internationally and has struggled to match passenger demand with available aircraft and crews, especially while adhering to SARS-CoV-2 mitigation strategies.

Aviation industry challenges over the last few years have occurred in an unexpected and juxtaposed relation to the positive progress seen in the space travel industry. During the pandemic, commercial space travel experienced many groundbreaking events to include the Virgin Galactic Unity-22, Blue Origin NS-16, and SpaceX Inspiration4 flights that took huge strides forward in industry development and captured worldwide interest in the prospect of future civilian space travel. This occurred during a period when many either chose not to fly or were unable to use airline travel due to pandemic-related impacts.

While scientific literature in aerospace medicine has always included concerns for international aviation as a vector for the carriage of disease, the reality of pandemic impacts over the last few years has highlighted this critical area of research. As expected, SARS-CoV-2 impacts related to flight has become a focus for aerospace health and the Research Topic received many submissions in this area. Investigators previously evaluating the complex biological interactions related to variable exposures encountered in the flight environment added SARS-CoV-2, and worked to better elucidate relations and health outcomes (Toprani et al.). Reports focused on clarifying international quarantine guidance for commercial aircrew (Vuorio et al.) and the significance of mental health concerns for aircrew related to isolation requirements.

Similar to pandemic-related impacts in the general population, research into mental health impacts associated with SARS-CoV-2 in aviators has emerged as a critical area for investigation with researchers evaluating the importance of assessing pilots for potential self-harm [Vuorio and Bor (a)] and suicide risk [Vuorio and Bor (b)]. Work has also been done to characterize the psychological impacts of job instability and insecurity upon aircrew associated with pandemic-related work restrictions (Görlich and Stadelmann). Outside of the SARS-CoV-2 focus, researchers continue to evaluate mental health factors in aviation, with the topic reviewing impacts of emotional dysregulation in pilots and the potential for increased risk taking approaches that could negatively affect safety of flight (Luciani et al.).

Given the regularly expected circadian impacts related to time zone change and shift work that is unavoidable in the aviation industry, work continues in evaluating health concerns with focus on the interplay between effects of both sleep and specific work exposures in pilots (Jun-Ya and Rui-Shan). Additionally, Wingelaar-Jagt et al. provided an extensive review of risk, prevention measures and available pharmacological interventions to mitigate fatigue effects in aviation. Medical advances continue to drive increases in the average age and the number of health conditions for the traveling general population, and research is helping to understand the impacts of in-flight effects, with investigations reporting potentially increased risk of hypoxia in older adult passengers (Meyer et al.), as well as work evaluating the types and numbers of ground-based vs. in-flight airport medical events which could help to inform the appropriate medical capabilities needed at air terminals (Lo et al.).

Alternatively, many other research concerns related to the flight environment occur due to exposures in high-performance flight, specifically in military application. Kelley et al. reported on mild pulmonary function changes observed in subjects exposed to conditions simulating military on-board oxygen delivery systems, while Yang Y. et al. noted increased risk of lower back pain in military pilots associated with high weekly flight hours, neck pain, weak abdominal muscle activation and reduced hip extensor/rotator strength. Finally, an extensive review by Scheibler et al. summarized the literature evidence of cancer in military and commercial aircrew related to cosmic radiation, reporting increased risk for melanoma and non-melanoma skin cancer, breast cancer and potentially brain cancer, however noting the epidemiological literature provides less evidence directly linking health effects to cosmic radiation vs. exposure to the multifactorial flight environment.

As discussed previously, interest into space travel has ballooned over the last few years, and similarly the topic experienced a surge in manuscripts focused on the space environment. Many articles investigated longstanding key areas of research including neurovestibular challenges, musculoskeletal/bone impacts and cardiovascular effects. Topic researchers evaluated repetitive transcranial magnetic stimulation as a therapy to mitigate gait disorders associated with simulated microgravity (Yang J. et al.) and reported on a novel sensorimotor training paradigm that could improve post-spaceflight neurovestibular deteriorations, thereby potentially enhancing human operational functioning upon arrival following long duration space travel (Ren et al.).

Researchers also worked to clarify bone cellular pathway responses caused by mechanical unloading as experienced in the microgravity environment (Zhao et al.) and reviewed the current risk exposures, injury mechanisms, health effects and validated countermeasures to mitigate musculoskeletal injuries in the high performance aviation and space environments (O'Conor et al.). Investigation into cardiovascular effects of space travel included assessment of lower body negative pressure training as an exercise countermeasure for orthostasis experienced in the microgravity environment (Parganlija et al.) and comparison of micro and macrocirculatory changes in response to microgravity experienced during parabolic flight to help elucidate cardiovascular effects of the space (Bimpong-Buta et al.). Huff et al. also provided a substantial review of space-related radiation risk along with an evaluation of existing cardiovascular disease clinical risk prediction models for use in the context of space travel, and proposed a framework for personalized space radiation risk assessment in NASA astronauts. The recent pivot to develop longer duration space travel in the not so distant future has pushed researchers to consider new areas including gastrointestinal health and changes related to long-term restriction of dietary options. Dong et al. evaluated fecal bile acid profiles in subjects consuming space rations in a spacecraft analog study and found evidence of individual response variation to dietary and environment changes, suggesting a personalized approach to metabolic and dietary design may be a component of future spaceflight planning. The impact of individualized gut microbiome variation in response to the space environment was also discussed in a commentary by Mikelsaar and Mändar that reviewed historical analysis of cosmonaut gastrointestinal samples. Finally a few manuscripts used a more general approach to discuss challenges of long duration deep space travel, including a review of physiologic and circadian changes expected to be encountered in deep space as travelers move farther away from the protections of earth's geomagnetic field and transition into the hypomagnetic environment of outer space (Xue et al.). Additional contributions included Sobel and Duncan evaluating major environmental health exposures in long duration spaceflight and briefly proposing potential countermeasures, and Criscuolo et al. providing a perspective on some of the economic, technological and philosophical questions raised in the development of plans for future deep space travel.

Scientific and regulatory forums continue work to better understand health risks for professional aviators and astronauts, as well as the traveling public. In the context of space travel, professional space organizations such as NASA have been successful in mitigating health risks in highly screened and trained astronauts, however the space environment poses new challenges related to the underlying health problems experienced by the general population. Currently most researchers agree the most pressing areas for investigation include space motion sickness, space-related psychological effects, acceleration forces and microgravity, cardiovascular responses and fluid shifts, bone and muscle loss, and spaceflight-associated neuro-ocular syndrome. Past efforts have been made to establish potential guidelines focused on health risk screening for commercial space passengers and spaceflight participants led by the Aerospace Medical Association (3), Federal Aviation Agency (4, 5) and the International Academy of Astronautics (6). In addition to stratifying medical risks, there have also been steps to determine regulatory requirements such as space crew qualification training and spaceflight participant risk counseling requirements (7), as well as commercial human spaceflight occupant safety standards (8). The recent advent of commercial civilian space travel occurring without validated participant screening and safety requirements highlights the importance of studying the data and passenger experiences from these initial events to inform future development and traveler protections (9).

Given the various commercial spaceflight providers this effort will need to be collaborative involving industry, government and academia, and will need to navigate many logistical and legal challenges such as safeguarding proprietary information of spaceflight providers. One potential avenue for this collaboration may be under the framework of a human research program (10). Given these broad areas requiring focused research and investigation into the space environment and the return of aviation travel to pre-pandemic levels, the field of aerospace health should continue to receive growing attention over the next decade.

## Author contributions

CS: Writing—original draft, Writing—review and editing. MC-G: Writing—review and editing. EM: Writing—review and editing.
